# Thomas W. Clements, D.D.S., D.M.D.

**Published:** 1913-12-15

**Authors:** H. A. Baker, F. S. Belyea, A. R. Brown

**Affiliations:** Committee; Committee; Committee


### THOMAS W. CLEMENTS, D.D.S., D.M.D.

The committee appointed to take action on the death of Dr. T. W. Clements begs to recommend that the Academy set aside a page of its records, In Memoriam, and that on the page be written the following:

In memory of our late Fellow, Thomas W. Clements, D.D.S., D.M.D.

He was a faithful soldier of the Republic, a good citizen, a true friend.

He exemplified to an extraordinary degree all those qualities, which in their sum, make the gentleman.

We mourn the loss of one who brought to our profession the richest gifts of mind and heart.

As an Ethical Practitioner, Teacher, College Trustee, Member and Officer in many Dental Societies, he did much for the advancement of Dentistry.

We sympathize with his many friends in their loss and we rejoice that it was our privilege to call him our friend. We are glad that such a high character and delightful personality came into the Profession of Dentistry.

H. A. BAKER,
F. S. BELYEA,
A. R. BROWN,
*Committee.*

